# Adapting to UV: Integrative Genomic and Structural Analysis in Bacteria from Chilean Extreme Environments

**DOI:** 10.3390/ijms26125842

**Published:** 2025-06-18

**Authors:** Mauricio Núñez, Antonia Naciff, Fabián Cuadros, Constanza Rojas, Gastón Carvallo, Carolina Yáñez

**Affiliations:** 1 Laboratorio de Microbiología, Instituto de Biología, Facultad de Ciencias, Pontificia Universidad Católica de Valparaíso, Valparaíso 234000, Chile; 2 Laboratorio de Ecología Vegetal, Instituto de Biología, Facultad de Ciencias, Pontificia Universidad Católica de Valparaíso, Valparaíso 234000, Chile; 3 Instituto de Ecología y Biodiversidad, IEB, Santiago 7800020, Chile

**Keywords:** extremophilic bacteria, UV resistance, DNA repair, photolyase, pigment biosynthesis, sporulation

## Abstract

Extremophilic bacteria from extreme environments, such as the Atacama Desert, Salar de Huasco, and Antarctica, exhibit adaptations to intense UV radiation. In this study, we investigated the genomic and structural mechanisms underlying UV resistance in three bacterial isolates identified as *Bacillus velezensis* PQ169, *Pseudoalteromonas* sp. AMH3-8, and *Rugamonas violacea* T1-13. Through integrative genomic analyses, we identified key genes involved in DNA-repair systems, pigment production, and spore formation. Phylogenetic analyses of aminoacidic sequences of the nucleotide excision repair (NER) system revealed conserved evolutionary patterns, indicating their essential role across diverse bacterial taxa. Structural modeling of photolyases from *Pseudoalteromonas* sp. AMH3-8 and *R. violacea* T1-13 provided further insights into protein function and interactions critical for DNA repair and UV resistance. Additionally, the presence of a complete violacein operon in *R. violacea* T1-13 underscores pigment biosynthesis as a crucial protective mechanism. In *B. velezensis* PQ169, we identified the complete set of genes responsible for sporulation, suggesting that sporulation may represent a key protective strategy employed by this bacterium in response to environmental stress. Our comprehensive approach underscores the complexity and diversity of microbial adaptations to UV stress, offering potential biotechnological applications and advancing our understanding of microbial resilience in extreme conditions.

## 1. Introduction

Extremophile microorganisms thrive in conditions that are considered inhospitable to most known life forms, such as extreme temperatures, high salinity, broad pH ranges, and UV radiation [1]. Their remarkable adaptability not only expands our understanding of the limits of life on Earth, but their study has also revealed notable adaptation mechanisms relevant to both biotechnology and astrobiology [2,3]. Ultraviolet (UV) radiation is one of the most challenging abiotic factors in environments with limited water availability and highly variable climates.

In Northern Chile, the Atacama Desert is recognized as one of the driest places on Earth, where the impact of UV radiation is especially severe due to low atmospheric moisture and high solar exposure [3,4,5]. In this environment, studies have identified bacteria from genera like *Microbacterium* and *Exiguobacterium* that exhibit remarkable UV resistance, achieved through molecular defense mechanisms and ecological strategies, notably the occupation of endolithic habitats and biological crusts that mitigate solar damage and retain moisture [6,7,8].

Beyond the extreme dryness of the Atacama Desert, the Chilean Altiplano is home to the Salar de Huasco, an environment characterized by the synergistic effects of high UV exposure, significant osmotic stress due to salinity, and scarce freshwater resources. Microorganisms inhabiting the Salar de Huasco have evolved diverse resistance strategies, which parallel those observed in other extreme habitats. These include the deployment of DNA-repair enzymes, detoxification of reactive oxygen species, synthesis of exopolysaccharides, and production of photoprotective compounds [7,9,10,11]. Such convergent evolution highlights the powerful selective forces of UV radiation and desiccation, leading to the development of similar molecular solutions across contrasting environments [12,13].

While the Atacama and Salar de Huasco experience UV stress due to aridity and salinity, Antarctica presents a different extreme: low temperatures and seasonal ozone depletion lead to UV radiation levels that surpass those found at lower latitudes [14]. Nonetheless, Antarctic bacteria have developed a robust set of adaptations to tolerate molecular damage, including efficient DNA-repair systems (photolyases, NER, and BER), antioxidant defenses, and the synthesis of photoprotective pigments [15,16,17].

In summary, from the hyperarid Atacama Desert to the hypersaline ecosystems of the Chilean Altiplano and the cold, irradiated landscapes of Antarctica, life faces formidable challenges such as UV radiation, desiccation, and osmotic stress [18]. However, nature has selected extremophilic microorganisms that have evolved DNA-repair mechanisms, antioxidant systems, and protective molecules, which serve as valuable models for biotechnological innovation and the search for life beyond Earth [3,5,13,19].

The main objective of this study was to perform a comprehensive genomic analysis of three extremophilic bacterial isolates obtained from different extreme environments: the Atacama Desert, the hypersaline salt flats of the Chilean Altiplano, and Antarctica. This analysis aimed to identify mechanisms of resistance to ultraviolet radiation. By comparing the genomes of these isolates, we sought to uncover common and distinct genetic adaptations that enable these microorganisms to survive in environments with high levels of UV radiation, extreme temperature fluctuations, and water scarcity.

Ultimately, this research aims to provide insights into the molecular pathways underlying UV resistance, with potential applications in biotechnology and astrobiology. Rather than pointing for broad generalization, this study focuses on an in-depth comparative genomic and structural analysis of three bacterial strains selected from highly contrasting UV-stressed environments in Chile. This approach seeks to generate hypotheses regarding UV-resistance mechanisms and evolutionary adaptation at the molecular level.

## 2. Results and Discussion

### 2.1. Genome Assembly and ANI-Based Taxonomic Assignment

**Figure 1:**
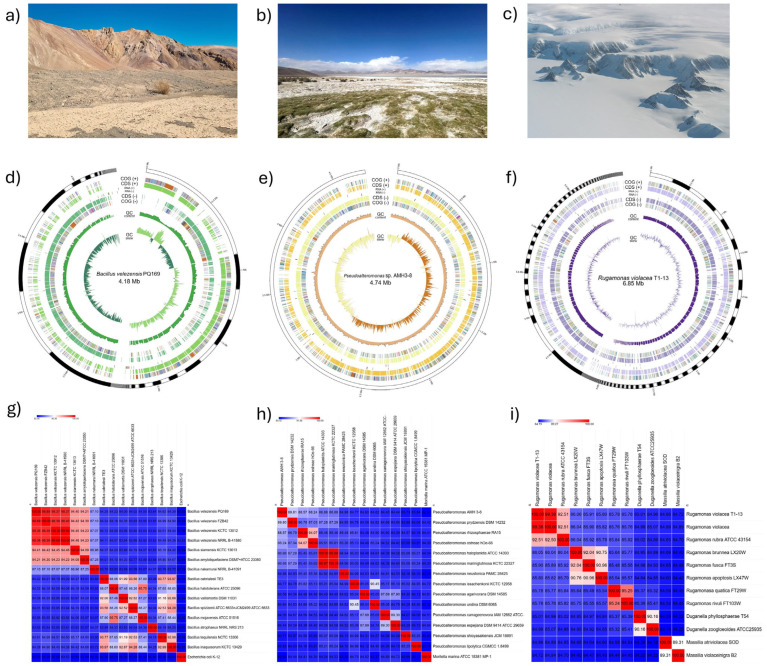
Isolation sites, genomic assembly characteristics, and ANI-based taxonomic assignment of three extremophilic bacterial isolates. (**a**–**c**) Representative sampling sites of bacterial isolates: (**a**) Atacama Desert, (**b**) Salar de Huasco, and (**c**) Antarctic soil. (**d**–**f**) Circular genome representations illustrating variability in genome size, contiguity, and genetic content: (**d**) *Bacillus velezensis* PQ169 (76 contigs, 4.18 Mb), (**e**) *Pseudoalteromonas* sp. AMH3-8 (single contig, 4.74 Mb), and (**f**) *Rugamonas violacea* T1-13 (171 contigs, 6.85 Mb). Rings (outer to inner) represent predicted CDSs, RNA genes (rRNA, tRNA, and tmRNA), GC content, and GC skew. (**g**–**i**) Heatmaps depicting average nucleotide identity based on MUMmer (ANIm) analysis between each isolate and closely related reference strains, supporting definitive taxonomic assignment: (**g**) *Bacillus velezensis* PQ169, (**h**) *Pseudoalteromonas* sp. AMH3-8, and (**i**) *Rugamonas violacea* T1-13. Color gradients indicate similarity percentages, with red color representing higher ANI values, and blue lower values.

The Microbiology Laboratory at Pontificia Universidad Católica de Valparaíso provided three extremophilic bacteria that were selected for this study, each isolated from a unique extreme environment. Specifically, strain PQ169 was recovered from the phyllosphere of the endemic cactus *Eriosyce aurata* at the Atacama Desert, strain AMH3-8 from the rhizosphere of *Carex misera* in the Salar de Huasco (Chilean Altiplano), and strain T1-13 from a topsoil sample collected near Arturo Prat Base in Antarctica (Figure 1, panels (a–c), respectively). Initially, the taxonomic identities of these strains were uncertain. To resolve this ambiguity, we performed genome sequencing and comparative genomic analyses.

Genome assembly revealed substantial differences in genome size, contiguity, and gene content among these isolates (Figure 1, panels (d–f)). The genome of isolate PQ169 comprised 76 contigs with an N50 of 336.008 bp, L50 of 4, and a total genome size of 4.18 Mb, encoding 4070 predicted coding DNA sequences (CDSs), 11 ribosomal RNA (rRNA) genes, 84 transfer RNA (tRNA) genes, and 1 transfer-messenger RNA (tmRNA). Isolate AMH3-8 exhibited a more contiguous assembly into a single contig, an N50 of 65.452 bp, L50 of 24, and a genome size of 4.74 Mb, containing 4114 CDSs, 4167 total annotated genes, 1 rRNA gene, 51 tRNAs, 1 tmRNA, and 2 repeat regions. Conversely, isolate T1-13 presented 171 contigs with a with an N50 of 91.810 bp, L50 of 21, and a total genome size of 6.85 Mb, harboring 5751 CDSs, 3 rRNA genes, 76 tRNAs, and 1 tmRNA gene.

These genomic variations likely reflect the distinct evolutionary histories and specific adaptations to their respective environmental niches [12]. An integrated genomic strategy was implemented to accurately assign taxonomic identities to these isolates. Initially, AutoMLST analysis was used to identify the closely related type strains. Following this, average nucleotide identity was based on MUMmer (ANIm) analyses conducted by aligning isolate genomes against selected reference strains. Additionally, digital DNA-DNA hybridization (dDDH) analyses were carried out using the Type (Strain) Genome Server (TYGS) platform, providing further taxonomic resolution.

ANIm analysis revealed that isolate PQ169 exhibited 98.89% similarity with *Bacillus velezensis* FZB42, with a DDH similarity of 91.3% relative to *Bacillus amyloliquefaciens* subsp. *plantarum* FZB42. Considering recent evidence confirming *B. velezensis* as a valid species distinct from *B. amyloliquefaciens* [20,21], this isolate was conclusively identified as *Bacillus velezensis* PQ169 (Figure 1g).

For isolate AMH3-8, the ANIm similarity was 89.81% with *Pseudoalteromonas prydzensis* DSM 14232, and DDH reached only 54.1%. Both values fell below established species delineation thresholds (ANI ≥ 97%, DDH ≥ 70%), suggesting isolate AMH3-8 represents a novel species within the genus *Pseudoalteromonas*. Moreover, its terrestrial origin notably contrasts with previously described *Pseudoalteromonas* species, typically isolated from marine environments [22]. Consequently, this isolate was designated as *Pseudoalteromonas* sp. AMH3-8 (Figure 1h).

Finally, isolate T1-13 showed a high genomic similarity (ANIm, 99.38%; DDH, 95.6%) to *Rugamonas violacea* CCM 8940, exceeding species-level thresholds. Thus, isolate T1-13 was confidently classified as *Rugamonas violacea (*Figure 1i). Notably, this represents the first reported isolation of *R. violacea* from soil, as opposed to the freshwater origin of the type strain (James Ross Island) [23], thereby broadening the ecological understanding of this bacterial species.

### 2.2. Gene-Content Analysis and DNA Repair-Pathway Components

After establishing the taxonomic identities and genomic structures of the three extremophilic isolates, we investigated their genomic content to elucidate the molecular mechanisms underpinning their UV resistance. Specifically, we focused on identifying genes encoding proteins associated with DNA-repair pathways involved in responding to UV-induced DNA damage. A comparative genomic analysis was conducted for 14 key DNA-repair proteins [24,25] across *Bacillus velezensis* PQ169, *Pseudoalteromonas* sp. AMH3-8, and *Rugamonas violacea* T1-13 (Table 1). The presence or absence of these proteins among the isolates may reflect differences related to their phylogenetic divergence and adaptation to their distinct environmental niches, providing deeper insight into bacterial strategies for coping with intense UV radiation.

All three strains encode the canonical components of the nucleotide excision repair (NER) system: UvrA, UvrB, UvrC, and the helicase UvrD. This complete set suggests that NER is a core mechanism shared among the isolates to remove bulky lesions such as cyclobutane pyrimidine dimers, one of the most common UV-induced DNA damages [26,27,28].

Photoreactivation, a direct repair mechanism, utilizes photolyase (*phr*), which was found in *Pseudoalteromonas* sp. AHM3-8 and *R. violacea* T1-13, but not in *B. velezensis* PQ169. This absence might indicate that *B. velezensis* compensates through enhanced excision or recombination-based repair, or it may reflect a different regulation of light-dependent repair systems in soil bacteria [29,30,31,32].

Regarding the SOS response, the recombinase RecA and the repressor LexA were conserved in all strains, implying that these bacteria are capable of sensing DNA damage and activating global repair responses [33]. The mismatch repair (MMR) proteins MutS and MutL were also consistently found, indicating that all strains possess mechanisms to correct replication errors and preserve genome stability [34].

Interestingly, MutT, which prevents the incorporation of oxidized guanine (8-oxo-dGTP), was not detected in *Pseudoalteromonas* sp. AMH3-8. This suggests that this strain may rely more heavily on post-replicative repair (e.g., via MutM and MutY) rather than on nucleotide pool sanitization [35]. In contrast, MutM and MutY, both central to base excision repair (BER) and essential for the recognition and removal of 8-oxoguanine lesions, were present in all three genomes [32,33,34,35].

The DNA polymerases PolB (DNA polymerase II) and DinB (polymerase IV), which allow translesion synthesis across damaged bases, also showed a variable pattern. PolB was absent in *B. velezensis* PQ169, possibly indicating alternative translesion polymerases or differing reliance on recombination-mediated gap repair [36,37,38].

Taken together, these findings show that all three isolates possess well-developed DNA-repair networks, with *R. violacea* T1-13 showing the most complete gene repertoire, which may reflect the combined selective pressures of UV, low temperatures, and oxidative stress in Antarctic environments. *Pseudoalteromonas* sp. AMH3-8 also shows a strong capacity for UV damage repair, despite lacking MutT. Meanwhile, *B. velezensis* PQ169 appears to depend more on core NER and BER mechanisms and lacks photoreactivation capability. This comparative analysis reveals both the conserved nature of essential DNA-repair systems and specific genomic adaptations that may reflect the environmental challenges faced by each strain. These results provide a genomic foundation for future experimental studies on the regulation, expression, and functional interplay of these systems in response to UV stress.

#### 2.2.1. Phylogenetic Analysis of the Nucleotide Excision Repair (NER) System

**Figure 2:**
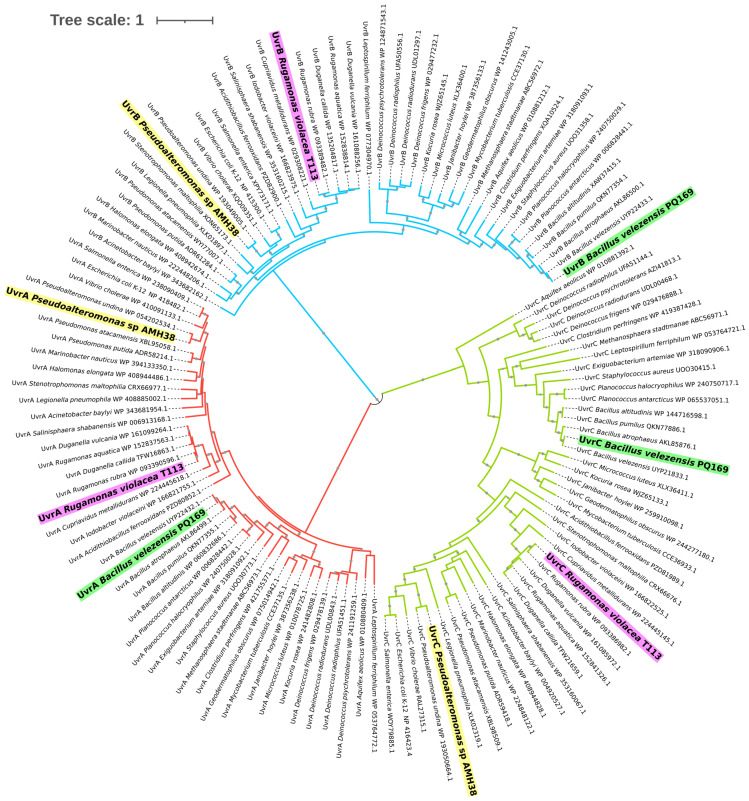
Phylogenetic tree based on the amino acid sequences of UvrA, UvrB, and UvrC proteins (NER system). Multiple sequence alignment was performed using MUSCLE on the Phylogeny.fr platform, followed by tree construction with PhyML and visualization using TreeDyn. The resulting tree was further arranged and refined using the Interactive Tree of Life (iTOL v7) tool. The maximum likelihood method was employed with 1000 bootstrap replicates, where the node sizes reflect the level of bootstrap support. Each label includes the gene name, the bacterial species, and the corresponding NCBI accession number. Three environmental strains are highlighted: *Bacillus velezensis* PQ-169 (green), *Pseudoalteromonas* sp. AMH3-8 (yellow), and *Rugamonas violacea* T1-13 (purple). These strains, along with other extremophilic, UV-resistant, and pathogenic bacteria, were included to compare UvrA (red clade), UvrB (blue clade), and UvrC (green clade) variations under extreme environmental conditions.

To gain a broader view of the NER system at the sequence level, we expanded our phylogenetic analysis to include amino acid sequences of UvrA, UvrB, and UvrC from a diverse panel of bacteria. UvrA initiates lesion recognition by scanning the DNA for damage, UvrB acts as a helicase verifying the damaged site, and UvrC provides endonuclease activity to excise UV-induced lesions [28]. Including these three components together allows for a more complete assessment of how the excinuclease ABC complex has evolved under different ecological and selective pressures [27,28,39]. Although each gene clusters into its own major branch corresponding to UvrA, UvrB, or UvrC, the same group of bacterial species was considered for each protein, enabling direct comparisons of their evolutionary trajectories under varying environmental pressures (Figure 2).

Across all three proteins, we observed a recurring pattern in which Gram-positive *Bacillus* species consistently grouped, reflecting strong selective constraints on DNA-repair functionality in taxa that endure desiccation, thermal fluctuations, and high UV exposure [40]. Halotolerant Gram-negative strains, such as *Pseudoalteromonas* sp. AMH3-8, formed a similarly cohesive set in each subunit’s cluster, suggesting that niche-specific adaptations, particularly those related to osmotic stress and UV tolerance, shape the evolution of the excinuclease system [39]. Pigmented and highly radiation-resistant bacteria, including *R. violacea* T1-13 and *Deinococcus* spp., appeared as distinct lineages within each major branch, indicating that protective pigments and robust DNA-repair pathways may coevolve to mitigate oxidative and UV-induced damage across phylogenetically diverse organisms [41,42,43].

Although key functional domains in UvrA, UvrB, and UvrC remain highly conserved, fine-scale sequence variations are apparent in isolates from extreme environments. This divergence is likely the result of convergent evolutionary pressures (e.g., intense solar radiation or extreme temperatures) that necessitate efficient and sometimes specialized DNA-repair mechanisms [39,41,44,45].

The fact that the same sets of strains occupy consistent positions in each Uvr subtree further underscores the importance of the excinuclease ABC system in supporting bacterial survival under harsh conditions. Taken as a whole, our findings demonstrate that while Uvr proteins retain a core functional architecture necessary for nucleotide excision repair, evolutionary modifications in each subunit may reflect fine-tuning to support DNA repair under environmental stressors found in deserts, polar regions, and hypersaline lakes. This comprehensive overview reinforces the value of our three environmental isolates, *B. velezensis* PQ169, *Pseudoalteromonas* sp. AMH3-8, and *R. violacea* T1-13, as comparative models for investigating how bacteria fine-tune the NER pathway to thrive in extreme and highly dynamic ecosystems.

These phylogenetic patterns suggest that, while the functional core of NER proteins is conserved, environmental pressures such as intense UV radiation, desiccation, and osmotic stress may drive adaptive divergence in flexible or regulatory domains. Such changes could affect DNA-damage recognition, protein–protein interactions, or expression dynamics, offering strain-specific optimization under distinct stress regimes. This supports the idea that the NER system, though functionally conserved, remains evolutionarily responsive to ecological challenges across extreme environments.

#### 2.2.2. Photolyases and Structural Insights

Photolyases are flavoproteins that repair UV-induced DNA lesions such as cyclobutane pyrimidine dimers (CPDs) and (6–4) photoproducts, relying on a conserved cryptochrome/photolyase (PHR) domain that binds flavin adenine dinucleotide (FAD) as a cofactor [16,25,46,47]. Absorption of light by FAD promotes an electron transfer reaction that restores the native DNA structure, thus maintaining genome stability [25]. While well-characterized in certain prokaryotic model organisms, photolyases are increasingly recognized across diverse bacterial species [15,48,49].

**Figure 3:**
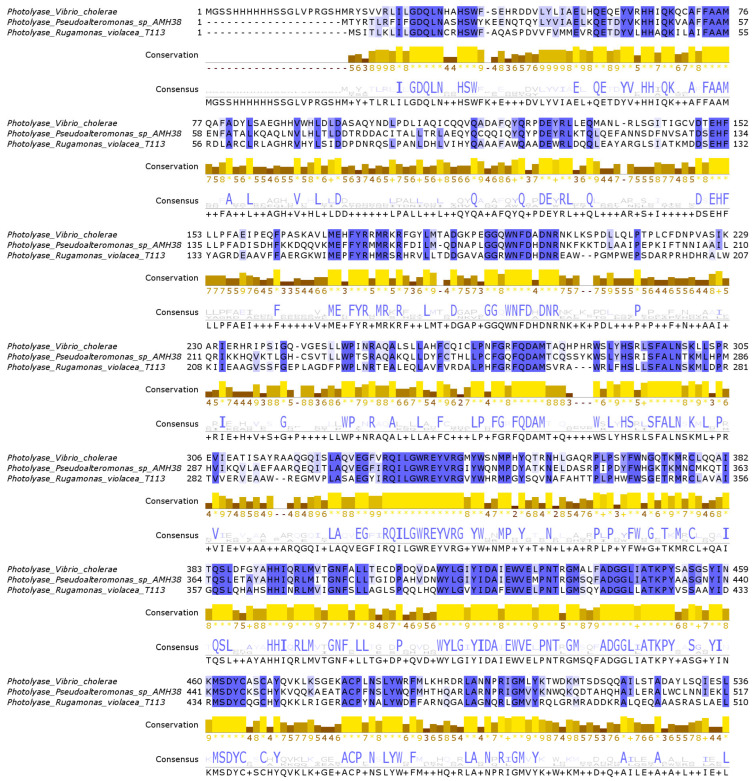
Multiple sequence alignment between the photolyase of *Vibrio cholerae* and photolyase-related proteins from *Pseudoalteromonas* sp. AMH3-8 and *Rugamonas violacea* T1-13. The alignment was generated in MEGA12, using the MUSCLE algorithm, and visualized in Jalview, where blue shading indicates residues with over 50% conservation. The consensus sequence (bottom line) and the associated histogram (in yellow/brown) further highlight highly conserved regions, supporting the structural and functional similarities observed among these photolyase-like proteins.

In the genomes of *Pseudoalteromonas* sp. AMH3-8 and *R. violacea* T1-13, we identified two genes that potentially encode deoxyribodipyrimidine photolyase-related proteins. To evaluate their sequence-level similarities, we generated a multiple sequence alignment (MSA) with *Vibrio cholerae* photolyase (PDB ID: 7YKN) as a reference (Figure 3). In this alignment, colored shading marks positions at or above 50% identity among the three sequences, while several sites display complete (100%) identity. These highly conserved residues are distributed across multiple regions of the alignment, reflecting the hallmark motifs of photolyases [50,51].

**Figure 4:**
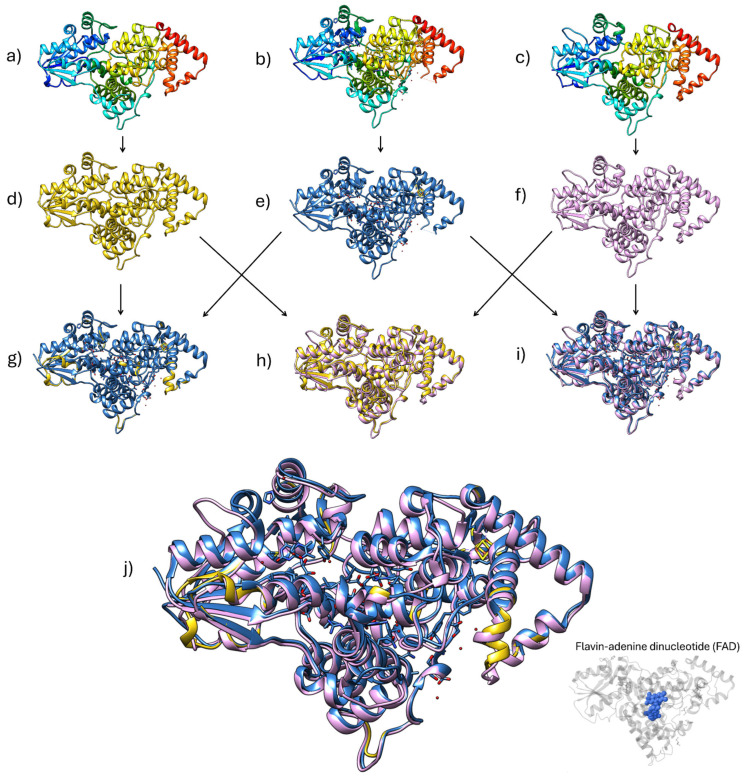
Comparative structural analysis of photolyase-related proteins from *Pseudoalteromonas* sp. AMH3-8, *Vibrio cholerae*, and *Rugamonas violacea* T1-13. (**a**) Three-dimensional (3D) model of the putative deoxyribodipyrimidine photolyase-related protein from *Pseudoalteromonas* sp. AMH3-8. (**b**) X-ray structure of the *Vibrio cholerae* photolyase (PDB ID: 7YKN), used as a reference in the structural comparisons. (**c**) Three-dimensional model of the photolyase-related protein from *R. violacea* T1-13. The recoloring (**d**–**f**) facilitates the identification of each model’s overall topology. (**g**) Superposition of the *Pseudoalteromonas* sp. AMH3-8 model and the *V. cholerae* photolyase, illustrating the high overall structural similarity. (**h**) Superposition of the *Pseudoalteromonas* sp. AMH3-8 and *R. violacea* T1-13 models, showing conserved folds. (**i**) Superposition of the *V. cholerae* photolyase and the *R. violacea* T1-13 model. (**j**) Combined overlay of all three structures (*Pseudoalteromonas* sp. AMH3-8, *V. cholerae*, and *R. violacea* T1-13), highlighting the putative FAD-binding pocket. These comparisons reveal a shared photolyase-like architecture among the three proteins, suggesting a conserved role in UV-induced DNA repair.

Subsequently, to gain insight into the structural relevance of these conserved residues, we performed three-dimensional (3D) modeling of the *Pseudoalteromonas* sp. AMH3-8 and *Rugamonas violacea* T1-13 photolyase-like proteins, using Phyre 2.2 and SWISS-MODEL, and compared the models to the crystal structure of the *Vibrio cholerae* photolyase (Figure 4). To further quantify the degree of structural similarity, we conducted pairwise Root Mean Square Deviation (RMSD) analysis, which underscores a highly conserved core domain pivotal to their putative biological function [52,53].

The comparison of *V. cholerae* photolyase and the *Pseudoalteromonas* sp. AMH3-8 model revealed a remarkably low pruned RMSD of 0.148 Å (492 atom pairs), which increased only slightly to 0.609 Å when all 505 equivalent positions were considered. This finding strongly suggests that *Pseudoalteromonas* sp. AMH3-8 shares an exceptionally well conserved folding pattern and active site architecture with *V. cholerae*, implying a near-identical arrangement of key residues crucial for FAD binding and photoreactivation.

In contrast, when *V. cholerae* was compared to the *R. violacea* T1-13 model, the pruned RMSD rose to 0.411 Å (432 atom pairs), and the global RMSD reached 2.138 Å (494 atom pairs). Although 0.411 Å still indicates a robust overlap in the core photolyase domain, the higher global RMSD suggests greater divergence in flexible loops or other peripheral regions [53]. A similar trend emerged from the comparison between *Pseudoalteromonas* sp. AMH3-8 and *R. violacea* T1-13, with pruned and global RMSDs of 0.444 Å (432 atom pairs) and 2.125 Å (503 atom pairs), respectively. These results imply that, while *R. violacea* T1-13 and *Pseudoalteromonas* sp. AMH3-8 maintain a core domain consistent with canonical photolyase structure, they likely differ somewhat in loop length or orientation relative to one another and to *V. cholerae*.

Despite these variations, all three proteins exhibit low RMSD values in their pruned (core) regions, indicating a high degree of structural conservation critical for the binding of the FAD cofactor and the facilitation of the electron transfer mechanism that underlies photoreactivation. Collectively, these observations reinforce the hypothesis that the *Pseudoalteromonas* sp. AMH3-8 and *R. violacea* T1-13 proteins preserve the fundamental fold and active site configuration characteristic of the photolyase family proteins [48,49]. Such close resemblance to a well-studied photolyase (*V. cholerae*) offers compelling evidence that both novel proteins likely retain the biological function of UV-induced DNA repair [15]. Moreover, given that photolyases have been shown to play pivotal roles in genomic maintenance and UV-stress responses, especially in environments with fluctuating UV exposure [16,48], these findings point to a potentially conserved photoreactivation pathway among these species. Nevertheless, further biochemical analyses, for instance, characterizing UV-induced DNA-repair activity in vitro, will be indispensable to confirm the functional predictions drawn from these in silico models.

Although the predicted structures exhibit high-confidence RMSD values and preserve conserved active site features, it is important to recognize the limitations inherent to in silico modeling. These predictions do not account for dynamic conformational changes, post-translational modifications, or context-dependent interactions in vivo. Therefore, future studies should focus on conducting gene expression analyses of these photolyase-like proteins and evaluating their enzymatic activity through in vitro UV-induced DNA-repair assays in order to experimentally validate their function and structural significance.

Furthermore, while the core architecture and FAD-binding domains of the modeled photolyases are highly conserved, the observed structural differences—particularly in loop regions and peripheral domains—may reflect functional specialization. These variations could modulate substrate affinity, DNA-binding kinetics, or interactions with auxiliary proteins involved in the UV-stress response. Although speculative, such divergence may result in differential repair efficiency or regulatory behavior under varying environmental conditions. Site-directed mutagenesis of these divergent regions, combined with in vitro photoreactivation assays, would be valuable for experimentally assessing the functional impact of the predicted structural variations.

### 2.3. Pigment-Based Mechanisms

**Figure 5:**
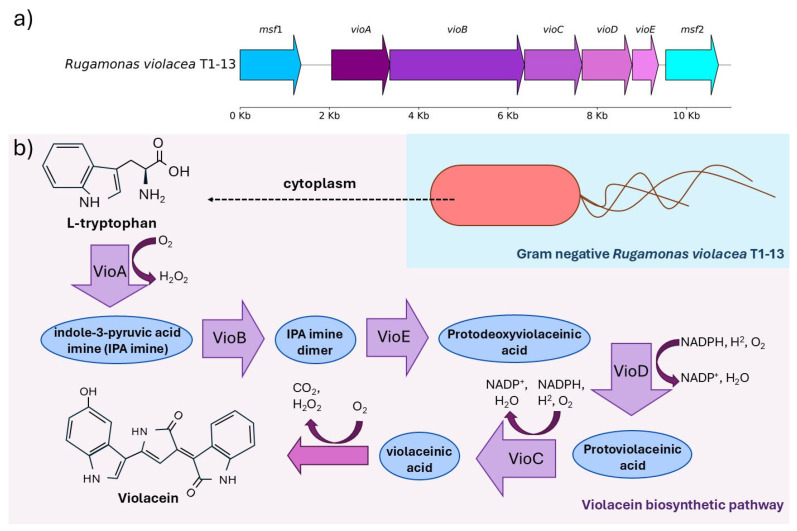
Genetic organization and biosynthetic pathway of violacein in *Rugamonas violacea* T1-13. (**a**) Genomic arrangement of the violacein operon in *R. violacea* T1-13, showing the sequential organization of key biosynthetic genes (e.g., *vioA*, *vioB*, *vioC*, *vioD*, and *vioE*) within a ~6 kb region. Different colored arrows indicate individual genes and their transcriptional orientation. (**b**) Proposed biosynthetic pathway for violacein production, starting from L-tryptophan and progressing through various intermediates (such as IPA imine and protodeoxyviolaceinic intermediates) to yield the final product, violacein. The pathway diagram also identifies the cytoplasmic localization of pigment biosynthesis in this Gram-negative bacterium.

Pigments play a crucial role in protecting cells against UV-induced damage by absorbing harmful wavelengths and scavenging reactive oxygen species, thereby contributing to an enhanced oxidative stress response. In our study, we identified a complete violacein operon in the genome of *Rugamonas violacea* T1-13, a bacterial isolate from the Antarctic environment. Notably, although we screened the genomes of the other two isolates for pigment-associated genes, we did not find a similarly organized gene cluster. Instead, only scattered genes potentially involved in pigmentation were detected. This suggests that *R. violacea* T1-13 possesses a specialized and coherent violacein biosynthetic pathway, which may confer enhanced photoprotective properties compared to the other strains. As detailed in Figure 5, a well-conserved gene cluster encodes the enzymes responsible for violacein biosynthesis. This operon structure is consistent with previously characterized violacein gene clusters in other bacteria, suggesting that *R. violacea* T1-13 can synthesize this pigment [23,54].

The stepwise conversion of L-tryptophan to violacein involves intermediate metabolites and highlights the sequential enzymatic reactions mediated by the *vio* gene products. The presence of a complete and organized violacein biosynthetic pathway supports the hypothesis that *R. violacea* T1-13 leverages pigment production as a protective mechanism against high levels of UV radiation commonly encountered in its native Antarctic habitat. Given violacein’s known antioxidant properties and its ability to absorb UV light, it is plausible that this pigment not only contributes to the cellular defense against UV-induced DNA damage but also to mitigating oxidative stress in extreme environments [17]. Collectively, these findings extend our understanding of the multifaceted UV-resistance strategies in extremophilic bacteria by illustrating how pigment biosynthesis integrates with other DNA-repair and stress-response pathways to ensure survival under adverse environmental conditions [17,55,56]. Future studies involving biochemical assays to confirm violacein production and its protective efficacy under UV stress will be critical to fully elucidate its role within the broader context of environmental adaptation in these microorganisms.

### 2.4. Sporulation as an Adaptive Response

**Figure 6:**
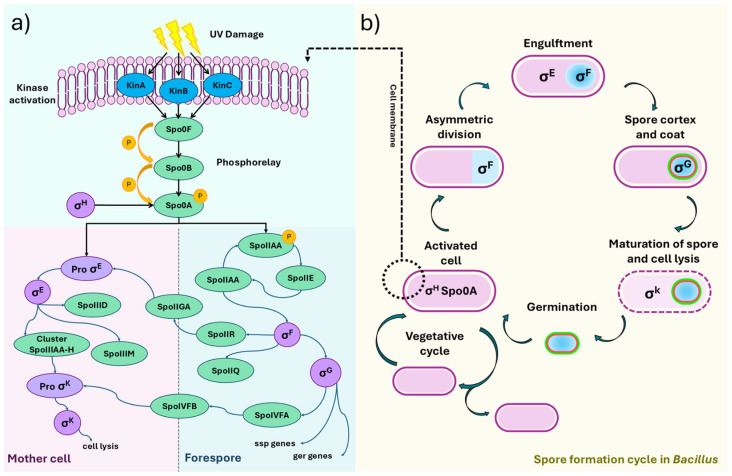
Spore-formation cycle in *Bacillus* due to UV damage. (**a**) Activation of the UV damage-response pathway at the membrane, where kinases KinA, KinB, and KinC are activated, leading to the phosphorylation of Spo0A. This process triggers the differentiation of the mother cell and the forespore. (**b**) Stages of spore formation, including asymmetric division; engulfment of the forespore by the mother cell; and the roles of σ^F^, σ^E^, σ^G^, and σ^K^ in spore maturation and the eventual lysis of the mother cell, leading to spore release and germination.

Endospore formation (sporulation) represents one of the most efficient bacterial strategies to cope with severe environmental stresses, including high levels of UV radiation [31]. In our genomic analysis of *Bacillus velezensis* PQ169, we identified the complete genetic machinery involved in sporulation (Figure 6), which is in line with the prediction that this mechanism may be important for adaptation and survival in the highly irradiated Atacama Desert environment [4].

Sporulation initiation in *Bacillus* spp. typically occurs upon sensing stressful conditions, such as nutrient limitation or DNA damage induced by UV radiation [57]. In *B. velezensis* PQ169, this process begins with sensor kinases (KinA, KinB, and KinC) located at the cell membrane, detecting environmental stress signals, illustrated here as UV radiation-induced DNA damage (Figure 6a). These kinases subsequently activate a phosphorelay system composed of Spo0F, Spo0B, and Spo0A, culminating in phosphorylation and activation of Spo0A, the master transcriptional regulator for sporulation.

Active Spo0A induces asymmetric cell division, generating a mother cell and a forespore (Figure 6b). Each compartment initiates a distinct transcriptional program controlled by specific sigma factors (σ). In the forespore, σ^F^ drives the early gene expression that is crucial for spore development, followed by σ^G^ activity, which regulates the late stages, including cortex and spore coat synthesis. The mother cell sequentially activates σ^E^ and σ^K^, coordinating the synthesis of protective layers around the developing spore, ultimately leading to cell lysis and spore release [58,59].

The structural integrity and biochemical resilience of bacterial spores are particularly relevant under UV stress. Spore formation involves the synthesis of UV-absorbing compounds (e.g., dipicolinic acid complexed with calcium ions), protective protein layers, and dehydration of the core, collectively conferring exceptional resistance to UV-induced DNA damage. These layers effectively shield genomic DNA from direct UV exposure, greatly diminishing photoproduct formation and subsequent DNA lesions [29,30].

In addition, recent studies have shown that sporulation in *Bacillus* is closely linked to RecA-mediated DNA-damage responses. RecA, beyond its canonical role in homologous recombination and SOS induction, plays regulatory and protective functions during sporulation. Activation of the SOS response depends on RecA and occurs in both the mother cell and forespore in response to UV and alkylation-induced DNA lesions, helping maintain genome integrity throughout development [60]. Furthermore, RecA has been implicated in modulating Spo0A phosphorylation levels during sporulation initiation and in repressing DNA replication at later stages, acting as a developmental checkpoint [32]. Importantly, inactivation of RecA or its accessory factors (RecF, RecO, RecR, and RecX) drastically reduces the survival of mature spores exposed to extreme stresses, such as ultrahigh vacuum desiccation and ionizing radiation, which cause single- and double-strand DNA breaks [61]. These findings underscore the essential role of RecA not only in stress-induced DNA repair but also in coordinating damage responses with sporulation progression and spore resilience.

Notably, the extensive genomic investment in sporulation-related genes observed in *B. velezensis* PQ169 suggests that spore formation is not merely an incidental protective measure, but a fundamental evolutionary adaptation to prolonged and intense UV radiation exposure characteristic of the Atacama Desert. This hypothesis aligns with previous observations in related *Bacillus* species, where sporulation has been confirmed as a primary adaptive response for survival under multiple environmental stresses, particularly desiccation and radiation [29,30,32,62]. Thus, our findings highlight the significance of the sporulation mechanism as a pivotal strategy in UV-stress resistance in *Bacillus velezensis* PQ169, underpinning the bacterium’s ecological success and persistence in one of Earth’s harshest environments.

Unlike previous studies that have focused on metagenomic surveys or on a single-model extremophile, this study provides an integrated genome level and structural characterization of UV resistance in three phylogenetically and ecologically distinct bacterial strains. By combining comparative genomics, gene-content profiling, structural modeling, and phylogenetic analyses, we provide new insights into how specific UV-resistance mechanisms may be shaped by environmental pressures such as desiccation, salinity, and temperature extremes. Furthermore, the inclusion of cultivated isolates from underexplored ecosystems such as the Chilean Atacama Desert, Altiplano, and Antarctic soils allows us to propose functional hypotheses grounded in isolate-level data, which can serve as a basis for future experimental validation and broader ecological comparisons.

While this study provides a comprehensive in silico characterization of UV-resistance mechanisms in three extremophilic bacterial strains, it is important to acknowledge its limitations. The predictions presented here, including gene annotations, structural models, and phylogenetic inferences, were derived from computational analyses and are not experimentally validated. Although the methodologies used are well established and supported by cross-validation (e.g., dual annotation pipelines and RMSD comparisons), functional confirmation of the proposed mechanisms remains necessary. Future work should include experimental validation of DNA-repair activity (e.g., through UV survival assays or gene knockouts); biochemical characterization of key proteins, such as photolyases; and transcriptomic analyses under UV stress to evaluate gene expression dynamics. These approaches will help test and refine the hypotheses generated in this study and strengthen our understanding of microbial adaptation to UV radiation in extreme environments.

## 3. Materials and Methods

### 3.1. Bacterial Isolates and Genomic DNA Extraction

The strains used in this study were previously isolated by the Microbiology Laboratory at the Pontificia Universidad Católica de Valparaíso. The geographic origins of the isolates were as follows: strain PQ169 from the Atacama Desert (27°6′36″ S, 69°43′37″ W), strain AMH3-8 from the Salar de Huasco (20°17′01″ S; 68°53′21″ W), and strain T1-13 from Antarctica (62°28′54″ S, 59°37′49″ W). Genomic DNA extraction was performed according to the protocol described by Chen et al. [63], with a minor adaptation for strain PQ169. Specifically, the cell pellet was pre-incubated with 25 µL of a 10 mg/mL lysozyme solution at 37 °C for 45 min before resuspension. DNA quality was assessed by agarose gel electrophoresis, and DNA concentration was measured using Qubit Fluorometer (Thermo Fisher Scientific Inc., Waltham, MA, USA).

### 3.2. Genome Sequencing, Assembly, Annotation, and ANIm-Based Taxonomic Analysis

For genome sequencing, the DNA were sent to TCL Group (Santiago, Chile) for library preparation and sequencing. DNA libraries were prepared using the MGIEasy FS DNA Library Prep Set and the MGIEasy Circularization Module V2.0 (MGI Tech, Tokyo, Japan). Sequencing was performed on the DNBSEQ-G400 platform using the DNBSEQ-G400RS High-Throughput Sequencing Set (MGI), with 150 bp paired-end reads to ensure high-quality, high-depth coverage. Quality controls were performed using FastQC v0.11.9 to assess the integrity and quality of the raw reads. Trimmed reads were generated using Trimmomatic v0.39 software. The genome assembly was then carried out de novo for all three isolates using Velvet v1.2.10. After assembly, genome annotation was performed with Prokka v1.14.6, which provided functional annotation of the assembled genomes. Circular genome maps were generated for each genome using GenoVI v0.2.16. For taxonomic assignment, an AutoMLST https://automlst.ziemertlab.com/ (accessed on 25 May 2025) analysis was performed to define the closest species based on core genome multilocus sequence typing. Next, an in silico hybridization was carried out using TYGS v400 (The Type Strain Genome Server) (https://tygs.dsmz.de/) to compare the genomes against available type strains. Finally, the average nucleotide identity (ANIm) was calculated using JSpeciesWS v4.3.2 (http://jspecies.ribohost.com/jspeciesws/). The ANIm approach that uses MUMmer (NUCmer) was used to align the input sequences. The ANIm graphical representation was generated using Morpheus https://software.broadinstitute.org/morpheus/ (accessed on 25 May 2025), which enabled the visualization and comparison of the ANIm values between the isolates and reference species. Additionally, the annotated genomes were uploaded to the RAST Server (Rapid Annotations using Subsystems Technology https://rast.nmpdr.org/ (accessed on 25 May 2025) to further explore gene functions and metabolic subsystems. This complementary approach allowed for cross-verification of annotation results obtained from Prokka and provided an interactive platform for visualizing and analyzing the predicted pathways and functional categories in each assembled genome.

### 3.3. Phylogenetic Analysis of UV Resistance-Related Proteins

Protein sequences of NER system (UvrA, UvrB, and UvrC) were retrieved from the National Center for Biotechnology Information (NCBI) database https://www.ncbi.nlm.nih.gov (accessed on 25 May 2025) according to their annotated gene names and accession numbers. The sequences were compiled into FASTA files, and multiple sequence alignments were performed in MEGA 12 (https://www.megasoftware.net), using the MUSCLE algorithm, with the following parameters: gap open = −2.90, gap extend = 0.00, hydrophobicity multiplier = 1.20, and UPGMA as the clustering method. When necessary, additional alignments were carried out on the Phylogeny.fr platform http://www.phylogeny.fr/ (accessed on 25 May 2025) and further refined in Jalview v2.11.4.1 (https://www.jalview.org). Phylogenetic trees were constructed under the maximum likelihood criterion with 1000 bootstrap replicates, with the relative bootstrap values indicating node support. For clarity and improved visualization, the resulting trees were uploaded and edited in the Interactive Tree of Life (iTOL v7) (https://itol.embl.de). Each sequence in the final tree is labeled with its respective protein name (UvrA, UvrB, or UvrC), bacterial species, and NCBI accession number.

### 3.4. Protein Structure Analysis

Proteins’ structural models (photolyases) were generated using SWISS-MODEL https://swissmodel.expasy.org (accessed on 25 May 2025) and Phyre 2.2 (https://www.sbg.bio.ic.ac.uk/phyre2), referencing the *Vibrio cholerae* photolyase (PDB ID: 7YKN https://www.ebi.ac.uk/pdbe/entry/pdb/7ykn) to guide template selection. Phyre predictions achieved 100% confidence for over 97% coverage of each query sequence. The predicted three-dimensional structures were visualized in UCSF Chimera v1.19 (https://www.cgl.ucsf.edu/chimera/), and selected models were further validated for structural consistency using the CONFORT tool integrated into the I-TASSER pipeline https://zhanggroup.org/I-TASSER/ (accessed on 25 May 2025). By combining these high-confidence structural predictions with sequence-based phylogenetics, we established a robust framework for examining both the evolutionary relationships and the potential functional implications of the photolyases in our isolates.

## 4. Conclusions

This study presents a comprehensive analysis of UV-resistance mechanisms in three extremophilic bacteria from distinct Chilean arid environments: highlands, desert, and Antarctica. Genomic and structural data revealed key adaptations, such as robust DNA-repair systems, photolyases, pigment biosynthesis, and sporulation, reflecting diverse strategies that may have been shaped by environmental UV stress. Phylogenetic analyses of NER components showed conserved evolutionary patterns, while structural modeling of photolyases in *Pseudoalteromonas* sp. AMH3-8 and *Rugamonas violacea* T1-13, along with the presence of a complete violacein operon in *R. violacea* T1-13, highlight functional adaptations to UV exposure. In *Bacillus velezensis* PQ169, a complete sporulation program supports endospore formation as a key survival mechanism in hyper-arid, high-irradiance conditions. Collectively, these findings deepen our understanding of microbial resilience under extreme radiation and reinforce the relevance of extremophiles as models in evolutionary biology and astrobiology.

## Figures and Tables

**Table 1:** Presence of key DNA-repair components related to UV-induced damage in the genome of three environmental bacterial strains: *Bacillus velezensis* PQ169, *Pseudoalteromonas* sp. AMH3-8, and *Rugamonas violacea* T1-13. Components are categorized according to their role in nucleotide excision repair (NER), base excision repair (BER), mismatch repair (MMR), direct photoreactivation, oxidative damage prevention, or the SOS response. A checkmark (✓) indicates the presence of the protein based on genome annotation, and a dash (–) denotes its absence or undetected annotation.

DNA-Repair Components	Function	*B. velezensis* PQ169	*Pseudoalteromonas* sp. AMH3-8	*R. violacea* T1-13
UvrA	Initiates nucleotide excision repair (NER) by recognizing DNA damage.	✓	✓	✓
UvrB	Participates in NER by verifying DNA damage and forming the incision complex.	✓	✓	✓
UvrC	Makes incisions on both sides of the DNA lesion in NER.	✓	✓	✓
UvrD	DNA helicase that removes the damaged DNA strand during NER.	✓	✓	✓
Phr	DNA photolyase that directly reverses UV-induced pyrimidine dimers using light energy.	–	✓	✓
RecA	Promotes homologous recombination and SOS response by facilitating LexA autocleavage.	✓	✓	✓
LexA	Repressor of SOS response; inactivated by RecA to induce DNA-repair genes.	✓	✓	✓
MutS	Mismatch recognition protein involved in mismatch repair (MMR).	✓	✓	✓
MutL	Acts as a matchmaker in MMR by coordinating repair proteins.	✓	✓	✓
MutT	Prevents incorporation of oxidized guanine (8-oxo-dGTP) into DNA.	✓	–	✓
MutM	Removes 8-oxoguanine from DNA via base excision repair (BER).	✓	✓	✓
MutY	Removes adenines misincorporated opposite 8-oxoguanine (G:A mismatch repair).	✓	✓	✓
PolB	DNA polymerase II involved in translesion synthesis and repair.	–	✓	✓
DinB	DNA polymerase IV, error-prone translesion polymerase involved in bypassing UV lesions.	✓	✓	✓

## Data Availability

The data presented in this study are available upon request from the corresponding author due to legal restrictions (intellectual property).
